# Incidence, indication and complications of postoperative reintubation after elective intracranial surgery

**DOI:** 10.1590/1516-3180.2013.1313440

**Published:** 2013-06-01

**Authors:** Lucas Yutaka Hayashi, Mariana Rodrigues Gazzotti, Milena Carlos Vidotto, José Roberto Jardim

**Affiliations:** I PT, MSc. Assistant Professor, Department of Physiotherapy, Centro Universitário São Camilo, São Paulo, Brazil.; II PT, PhD. Full Professor, Department of Physiotherapy, Centro Universitário São Camilo, São Paulo, Brazil.; III PT, PhD. Full Professor, Department of Physiotherapy, Universidade Federal de São Paulo (Unifesp), São Paulo, Brazil.; IV MD, PhD. Full Professor, Department of Pneumonology, Universidade Federal de São Paulo (Unifesp), São Paulo, Brazil.

**Keywords:** Ventilator weaning, Neurosurgery, Respiration, Intensive care units, Length of stay, Desmame do respirador, Neurocirurgia, Respiração, Unidades de terapia intensiva, Tempo de internação

## Abstract

**CONTEXT AND OBJECTIVE:**

There are no reports on reintubation incidence and its causes and consequences during the postoperative period following elective intracranial surgery. The objective here was to evaluate the incidence of reintubation and its causes and complications in this situation.

**DESIGN AND SETTING:**

Prospective cohort study, using data obtained at a tertiary university hospital between 2003 and 2006.

**METHODS:**

169 patients who underwent elective intracranial surgery were studied. Preoperative assessment was performed and the patients were followed up until hospital discharge or death. The rate of reintubation with its causes and complications was ascertained.

**RESULTS:**

The incidence of reintubation was 12.4%, and the principal cause was lowered level of consciousness (71.5%). There was greater incidence of reintubation among females (P = 0.028), and greater occurrence of altered level of consciousness at the time of extubation (P < 0.0001). Reintubated patients presented longer duration of mechanical ventilation (P < 0.0001), longer stays in the intensive care unit (ICU) and in the hospital (P < 0.0001), greater incidence of pulmonary complications (P < 0.0001), greater need for reoperation and tracheostomy, and higher mortality (P < 0.0001).

**CONCLUSION:**

The incidence of reintubation in these patients was 12.4%. The main cause was lowering of the level of consciousness. Female gender and altered level of consciousness at the time of extubation correlated with higher incidence of reintubation. Reintubation was associated with pulmonary complications, longer durations of mechanical ventilation, hospitalization and stay in the ICU, greater incidence of tracheostomy and mortality.

## RESUMO

**CONTEXTO E OBJETIVO:**

Não há relatos sobre incidência de reintubação, suas causas e consequências no pós-operatório de cirurgia intracraniana eletiva. O objetivo foi avaliar a incidência de reintubação, suas causas e complicações em pós-operatório de cirurgia intracraniana eletiva.

**TIPO DE ESTUDO E LOCAL:**

Estudo de coorte prospectivo, com dados que foram obtidos de 2003 a 2006 em um hospital universitário terciário.

**MÉTODO:**

169 pacientes submetidos a cirurgia intracraniana eletiva foram estudados. Foi realizada avaliação pré-operatória e os pacientes foram acompanhados até a alta hospitalar ou óbito, verificando a taxa de reintubação, suas causas e complicações.

**RESULTADOS:**

A incidência de reintubação foi de 12,4% sendo a principal causa o rebaixamento do nível de consciência (71,5%). Houve maior incidência de reintubação no sexo feminino (P = 0,028), bem como do nível de consciência alterado no momento da extubação (P < 0,0001). Pacientes reintubados apresentaram maior tempo de ventilação mecânica (P < 0,0001) e de internação em unidade de terapia intensiva (UTI) e hospitalar (P < 0,0001), maior incidência de complicações pulmonares (P < 0,0001), maior necessidade de reoperação e traqueostomia, além de aumento de mortalidade (P < 0,0001).

**CONCLUSÃO:**

A incidência de reintubação nesses pacientes foi de 12,4%. A principal causa da reintubação foi o rebaixamento do nível de consciência. O sexo feminino e nível de consciência alterado no momento da extubação foram relacionados à maior incidência de reintubação. A reintubação foi associada a complicações pulmonares, maior tempo de ventilação mecânica, de internação e de permanência na UTI, maior incidência de traqueostomia e mortalidade.

## INTRODUCTION

Interruption or discontinuation of mechanical ventilation is standardized according to evidence-based guidelines, but even with their use, some patients are unable to withstand spontaneous ventilation and need to be reintubated.^1^^,^^2^^,^^3^^,^^4^ The incidence of reintubation after discontinuation of mechanical ventilation ranges from 2% to 25% among clinical patients and from 0.3% to 4% among surgical patients, and the primary cause for reintubation is respiratory insufficiency.^5^^,^^6^^,^^7^^,^^8^^,^^9^

Patients who need reintubation present higher rates of mechanical ventilator-associated pneumonia, longer stay in intensive care units (ICUs) and longer hospital stay.^1^^,^^6^^,^^10^^,^^11^^,^^12^ Neurosurgical procedures such as intracranial operations have been reported to be a significant risk factor for the development of pulmonary complications.^4^ However, these complications have not been correlated with patient reintubation, nor are there any reports on patient reintubation during the postoperative period following elective intracranial neurosurgery.

Therefore, this lack of information justified the present prospective study to determine the incidence, causes and complications among patients under postoperative care following elective intracranial neurosurgery.

## OBJECTIVE

The objective was to evaluate the incidence of reintubation and its causes and complications during the postoperative period following elective intracranial surgery.

## METHODS

This was a prospective cohort study in which all patients in the neurosurgical ICU of Hospital São Paulo, Universidade Federal de São Paulo (Unifesp), participated between July 2003 and July 2006. This study included: 1) candidates for elective surgery to treat intracranial tumors, aneurisms, arteriovenous malformations, Arnold-Chiari disease, intracranial hematomas and abscesses; 2) patients who maintained spontaneous ventilation at the preoperative evaluation; 3) patients who had undergone surgery under general anesthesia; and 4) patients in the neurosurgery ICU during the postoperative period who were on mechanical ventilation at admission.

The exclusion criteria were 1) death before surgery; 2) tracheostomy performed before weaning from mechanical ventilation; and 3) unplanned extubation. This study was approved by the Research Ethics Committee of Hospital São Paulo, Universidade Federal de São Paulo, and an informed consent statement was obtained from all the patients.

Continuous variables were analyzed using the independent Student t and Mann-Whitney tests. Categorical values were analyzed using the chi-square test and Fisher’s exact test, with the level of significance set at 5% in all cases.

### Preoperative evaluation

During the preoperative period, all the patients answered a questionnaire that included questions asking for information on dyspnea, coughing, bronchospasm and smoking. The physical examination included observation of the type of cough and sputum; pulmonary auscultation; chest assessment; measurement of the level of consciousness using the Glasgow Coma Scale (GCS); identification of any presence of prior pneumopathy; presence of any preexisting diseases such as arterial hypertension, cardiopathy and diabetes mellitus; existence of any prior intracranial surgery; identification of the diagnosis and site affected; and risk classification by means of the appraisal of self-care agency (ASA) scale.

After the surgical procedure, the patients were sent to the ICU and those who were still intubated remained on mechanical ventilation. Postoperatively, the patients were followed up every day by the same team as in the preoperative period, until they were discharged or died.

### Assessment of the possibility of discontinuation of mechanical ventilation

The patients underwent daily clinical and respiratory assessments to determine the possibility of discontinuation of mechanical ventilation, and those who presented satisfactory evaluations underwent the spontaneous breathing trial (SBT). A satisfactory daily evaluation was considered to include the following: (a) consent from the neurosurgeon responsible; (b) GCS > 8 T; (c) body temperature less than 38 °C; (d) no treatment with vasoactive drugs (dobutamine or dopamine was allowed at doses less than 5 mg/kg of body weight per minute); and (e) adequate gas exchange, as indicated by an arterial oxygen pressure (PaO2) of at least 60 mmHg, with an inspired oxygen fraction of less than 0.4 and positive end-expiratory pressure (PEEP) of no more than 5 cmH2O.

### Spontaneous breathing trial

The SBT lasted for between 30 and 120 min and was performed with a T tube or pressure support of less than 8 cmH2O and PEEP less than or equal to 5 cmH2O. The parameters used to define failure or unsuccessful SBT were the presence of two of the following criteria: (a) arterial oxygen saturation (SaO2) under 90%; (b) breathing rate greater than 35 breaths/min over a 10-minute period; (c) 20% decrease or increase in systolic arterial pressure; (d) presence of signs of increased respiratory work for more than 15 min; (e) sudoresis and agitation; and (f) low consciousness level (GCS < 8 T). At this time, the SBT was stopped and mechanical ventilation was restored with the previous ventilator parameters. Otherwiser, after successful conclusion of the SBT, patients were extubated after medical consent had been obtained.

### Criteria for extubation failure

Failure in extubation was considered to exist when the patients needed to be reintubated within 48 hours. The decision to reintubate the patient was based on clinical deterioration, as shown by at least one of the following criteria: decreased level of consciousness (GCS < 8); significant worsening of pH or arterial PaCO2; SaO2 lower than 90%, with inspired oxygen fraction over 0.5; and signs of increased respiratory work (tachypnea, use of accessory breathing muscles and paradoxical pattern).

### Level of consciousness at the time of extubation

The patients were divided into two groups based on their consciousness level at the time of extubation: GCS of 10-11 T and 8-9 T, in which the letter T shows that the patient had an orotracheal tube at the time of the evaluation. The normal group was considered to be those with GCS of 10-11 T, since they were capable of responding to simple commands, and the altered group was considered to be those with GCS of 8-9 T, since they did not show any logical response to simple commands.

### Causes of reintubation

The causes of reintubation were classified as respiratory insufficiency, upper airway obstruction, hemodynamic instability or lowered level of consciousness.

### Respiratory complications of reintubation

Complications of reintubation such as tracheobronchitis and pneumonia were monitored. Pneumonia was defined as the presence of pulmonary infiltrate on the chest X-ray, associated with at least two of the following signs: purulent tracheobronchial sputum, increased body temperature (over 38.3 °C), and leukocytosis (more than 25% above the baseline count).^12^

## RESULTS

**Figure 1.**
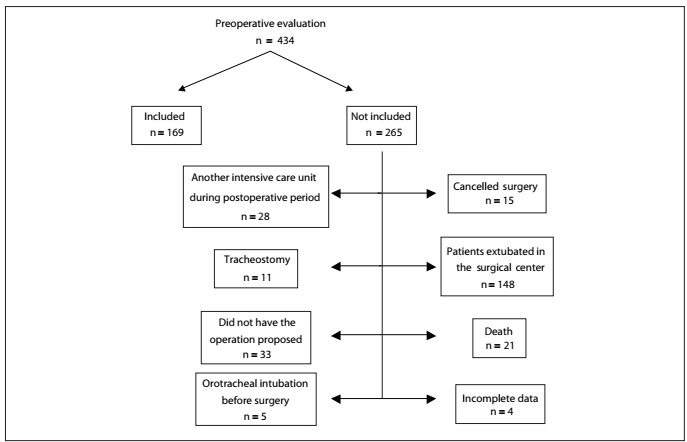
Organizational chart representing inclusion and exclusion of patients in the study.

Four hundred and thirty-four patients who had undergone intracranial surgery were hospitalized in the neurosurgical ICU during the study period, and 169 patients were included in the study. Among the 265 individuals who were excluded, 15 had their operations cancelled, 148 were extubated in the surgical center, 21 died before weaning off mechanical ventilation, 4 did not conclude the preoperative evaluation, 5 were ventilated before surgery, 33 did not have the operation proposed, 11 were tracheostomized and 28 were delivered to another ICU during the postoperative period (Figure 1).

The demographic characteristics of the patients included in the study are presented in Table 1. All of the 169 patients were weaned off mechanical ventilation and extubated, but 21 of these (12.4%) required reintubation within 48 hours. The causes of extubation failure were lowered level of consciousness in 71.5% of the cases, respiratory insufficiency in 19% and upper airway obstruction in 9.5%.

**Table 1.** Demographic characteristics of the 169 patients included in the study

Characteristics	Mean (SD) or n (%)
Age (years)	45.2 (15.1)
Female gender	91 (53.8%)
High surgical risk	15 (8.9%)
Respiratory symptoms	15 (8.9%)
Prior pulmonary disease	14 (8.2%)
Smokers	82 (4.5%)
Prior clinical disease	66 (39%)
Prior intracranial surgery	15 (8.8%)
Preoperative altered state of consciousness	6 (3.5%)
Diagnosis	
Tumor	108 (63.9%)
Cerebral vascular lesion	61 (36.1%)
Location of tumor	
Supratentorial region	133 (63.9%)
Infratentorial region	36 (21.3%)
Duration of surgery (min)	319.5 (90.1)
Altered level of consciousness at extubation	15 (8%)
Duration of MV before extubation (hours) median (25^th^-75^th^ percentile)	20 (11-31)
ICU LOS (days), median (25^th^-75^th^ percentile)	4 (2-9)
Hospital LOS (days), median (25^th^-75^th^ percentile)	9 (6-22)
Postoperative pulmonary complication before extubation	14 (8.2)
Postoperative pulmonary complication after extubation	38 (22.4)

MV = mechanical ventilation; ICU = intensive care unit; LOS = length of stay; SD = standard deviation.

Univariate analysis found three variables before extubation that differed between the patients who required reintubation and those who were successfully extubated: female gender, GCS at the time of extubation and duration of mechanical ventilation (Table 2). After extubation, seven other variables were identified as differing between the patients who needed reintubation and those successfully extubated: duration of mechanical ventilation, length of stay in the ICU, length of hospital stay, pulmonary complications, reoperation, tracheostomy and mortality (Table 3).

**Table 2.** Patient characteristics before extubation

Characteristics	Total number	Reintubation	No reintubation	P	Odds ratio	95% CI
Gender - n (%)						
Male	78 (46.2)	5 (6.4%)	73 (93.6%)	0.028*	3.1	[1.09;8.94]
Female	91 (53.8%)	16 (17.6%)	75 (82.4%)			
Age - median (SD)	45.2 (15.1)	48.8 (3.7)	44.7 (1.2)	0.479		
ASA - n (%)						
Low risk (n = 154)	154 (91.1%)	19 (12.3)	135 (87.7)	0.911	1.09	[0.23;5.22]
High risk (n = 15)	15 (8.9%)	2 (13.3)	13 (86.7)			
Respiratory symptoms - n (%)						
No (n = 150)	150 (88.8)	19 (12.6)	131 (87.4)	0.790	0.81	[0.17;3.79]
Yes (n = 19)	19 (11.2%)	2 (9.5)	17 (90.5)			
Prior pulmonary disease - n (%)						
No (n = 148)	148 (87.6%)	14 (9.5)	134 (90.5)	0.141	0.86	[0.81;0.92]
Yes (n = 21)	21 (12.4%)	0 (0)	21 (100)			
Smokers - n (%)						
No	86 (51.5%)	11 (12.8)	75 (87.2)	0.907	0.94	[0.38;2.36]
Yes	82 (48.5%)	10 (12.2)	72 (87.8)			
Prior clinical disease - n (%)						
No (n = 103)	103 (61%)	14 (13.6)	89 (86.4)	0.566	0.75	[0.29;1.98]
Yes (n = 66)	66 (39%)	7 (10.6)	59 (89.4)			
Prior intracranial surgery - n (%)						
No (n = 140)	140 (82.8%)	17 (12.1)	123 (87.9)	0.806	1.15	[0.36-3.73]
Yes (n = 29)	29 (17.2%)	4 (13.8)	25 (86.2)			
LC before surgery						
Normal	146 (86.4%)	128 (87.7)	18 (12.3)	0.923	1.06	[0.29-3.95]
Altered	23 (13.6%)	20 (87)	3 (13)			
LOS before surgery in days - mean (SD)		7.52 (9.15)	10.22 (8.89)	0.346		
Diagnosis						
Tumor (n = 108)	108 (63.9%)	13 (12)	95 (88)	0.838	1.10	[0.43;2.83]
Vascular lesion (n = 61)	91 (36.1%)	8 (13.1)	53 (86.9)			
Site of surgery						
Supratentorial (n = 133)	133 (63.9%)	16 (12)	117 (88)	0.764	1.17	[0.40;3.47]
Infratentorial (n = 36)	36 (21.3%)	5 (13.9)	31 (86.1)			
Duration of surgery in min - mean (SD)		302.1 (99.7)	322 (88.8)	0.304		
LC at extubation - n (%)						
Normal (n = 154)	154 (92%)	14 (9.1%)	140 (90.9%)	< 0.0001*	8.75	[2.76;27.73]
Altered (n = 15)	15 (8%)	7 (46.7%)	8 (53.3%)			
Duration of MV before extubation - median (25^th^ - 75^th^ percentile)		31.5 (13.75-124.50)	20 (11-28)	0.024*		
Postoperative pulmonary complication before extubation - n (%)						
No	157 (92.9%)	16 (76.1)	141 (95.2)	0.001*	6.29	[1.78:22.16]
Yes	12 (7.1%)	5 (23.9)	7 (4.8)			

LOS = length of stay; LC = level of consciousness; MV = mechanical ventilation;. *P < 0.05; SD = standard deviation; CI = confidence interval.

**Table 3.** Patient characteristics after extubation

Characteristics	Total number	Reintubation	No reintubation	P	Odds ratio	95% CI
Postoperative pulmonary complication after reintubation						
Absent	141 (83.4%)	9 (42.8)	132 (89.2)	< 0.0001*	11	[4.01;30.14]
Present	28 (16.6%)	12 (57.2)	16 (10.8)			
Reoperation - n (%)						
No (n = 152)	152 (89.9%)	11 (7.2%)	141 (92.8%)	< 0.0001*	18.31	[5.83;57.50]
Yes (n = 17)	17 (10.1%)	10 (58.8%)	7 (41.2%)			
Tracheostomy - n (%)						
No (n = 138)	159 (94.0%)	13 (8.2%)	146 (91.8%)	< 0.0001*	44.92	[8.62;233.92]
Yes (n = 179)	10 (6.0%)	8 (80%)	2 (20%)			
Deaths - n (%)						
No (n = 138)	153 (90.5%)	10 (46.6%)	143 (96.6%)	< 0.0001*	31.46	[9.13;108.31]
Yes (n = 179)	16 (9.5%)	11 (51.4%)	5 (3.4%)			
Total duration of MV - hours - median (25^th^-75^th^ percentile)	315	295 (76.5-353)	20 (11-28)	< 0.0001*		
ICU LOS - days - median (25^th^-75^th^ percentile)	27	24 (9.5-40)	3 (2-6)	< 0.0001*		
LOS in ward after discharge from ICU in days - median (25^th^-75^th^ percentile)	11	6 (0-30.5)	5 (3-9)	0.941		
Total LOS in days - median (25^th^-75^th^ percentile)	39	31 (12.5-59.5)	8 (6-17.75)	< 0.0001*		

MV = mechanical ventilation; ICU = intensive care unit; LOS = length of stay; CI = confidence interval. *P < 0.05.

There was no difference between those successfully extubated and those who were reintubated regarding the following: age, surgical risk assessed according to the ASA, respiratory symptoms, prior pulmonary disease, smoking, previous neurosurgery, length of hospital stay before surgery, diagnosis, anatomical encephalic site of the operation and duration of the operation.

## DISCUSSION

Approximately 12.5% of the patients included in the study needed reintubation, and the causes of extubation failure were lowered level of consciousness (71.5%), respiratory insufficiency (19%) and upper airway obstruction (9.5%). There were associations with female gender, GCS at the time of extubation and duration of mechanical ventilation. Reintubated patients presented longer duration of mechanical ventilation, longer stays in the ICU and in the hospital, and greater incidence of pulmonary complications, reoperation, tracheostomy and mortality.

The incidence of reintubation among clinical patients ranges from 2% to 25%, even in patients undergoing daily monitoring and SBT, which evaluate whether the individual is capable of being weaned off mechanical ventilation and extubated.^1^^,^^5^^,^^12^^,^^13^^,^^14^^,^^15^^,^^16^^,^^17^ Among surgical patients, the incidence of reintubation is lower, ranging from 0.3 to 4%.^7^^,^^8^^,^^9^ The incidence of reintubation among neurosurgical patients ranges from 16 to 35%.^19^ The conditions previously suffered by patients who were reintubated include: stroke, hypertensive intracerebral hemorrhage, subarachnoid hemorrhage, cranial-encephalitic trauma, encephalitis or metabolic encephalopathy; or the patients underwent spinal surgery. However, most of these patients had undergone emergency surgery, and a minority had undergone elective intracranial operations.^19^ In the present study, during the postoperative period following elective intracranial surgery, the incidence of reintubation was 12.4%. This rate is high in comparison with general elective operations, but lower than the prevalence of reintubation among neurosurgical patients, including non-elective operations.

Compared with elective surgery, this incidence seems too high, and therefore, perhaps, other criteria besides those already described in weaning guidelines should be developed and analyzed at the time of extubation of patients who have undergone elective neurosurgery. Noninvasive monitoring of the cerebral flow may be one of these. Many studies have shown that respiratory insufficiency is the primary cause of reintubation, among cases of both planned and unplanned reintubation.^8^^,^^9^^,^^18^^,^^20^^,^^21^^,^^22^^,^^23^

Nevertheless, we found that a lowered level of consciousness was the main cause of postoperative reintubation following elective intracranial surgery (71.5%). This may relate to the surgical procedure or to another factor relating to the respiratory center consequent to surgical manipulation, which would be likely to lead to hypoventilation and consequent CO2 elevation, along with a drop in the level of consciousness.

In a population of neurosurgical patients, Namen et al.^19^ studied the association between the level of consciousness and reintubation. They observed that a GCS score greater than or equal to eight was associated with successful extubation in 75% of the cases, whereas the proportion was only 33% among their patients with a GCS score lower than eight. Our study confirmed that patients with a low level of consciousness who had a GCS score less than or equal to nine at the time of extubation presented higher incidence of reintubation. In our study, we also noted that patients who had a longer duration of mechanical ventilation during the postoperative period presented higher incidence of reintubation.

This may relate to the surgical complexity or, on the other hand, to the alteration in consciousness level, which would explain the longer duration of mechanical ventilation. Mechanical ventilation for more than 48 hours may contribute towards pulmonary complications in patients who have undergone intracranial surgery,^24^ and to worsening of gas exchanges.^25^ These complications may also contribute towards reintubation. Reintubation is associated with increased hospital mortality, longer hospital stay, higher incidence of pneumonia, high hospital costs and a greater incidence of tracheostomy.^5^^,^^11^^,^^21^^,^^23^ In the same way, we observed that in the postoperative phase following elective intracranial surgery, our patients who were reintubated spent longer times on of mechanical ventilation (P < 0.0001), remained in the ICU for longer times (P < 0.0001), had longer hospital stays (P < 0.0001) and presented higher incidence of respiratory complications (P < 0.0001).

Mortality is another consequence of reintubation. Demling et al.^6^ observed a 40% mortality rate among surgical patients who were reintubated. Many studies have shown that the mortality rate associated with reintubation is 2.5 to 10 times higher than among patients who are not reintubated.^3^^,^^12^^,^^16^^,^^17^ In our study, we found a mortality rate of 51.4% among the patients who were reintubated, compared with 3.4% among patients who were not. Various hypotheses have been put forward to explain the association between reintubation and the high mortality rate: complications from reintubation, clinical deterioration between extubation and reintubation, ventilator-associated pneumonia, or the adverse effects from longer durations of mechanical ventilation.^26^ Additionally, the mortality among our patients may have been associated with complications and changes in the central nervous system, particularly the brain, due to operative manipulation and its complications.

Another factor that might have contributed towards the high mortality rate among our patients was the need for a new surgical approach. In our study, we noted that there was an association between reintubation and the need for a new surgical procedure (58.8%). Since the primary cause of reintubation was a reduced level of consciousness, another hypothesis that may explain the reduced level of consciousness would probably be the increased intracranial pressure, which may often be a reflex of problems secondary to surgery, thus requiring a new operation.

In this study, we did not evaluate any predictive index for weaning off mechanical ventilation, which may have had some association with the outcomes among these patients. Perhaps through assessment of neuroventilatory decoupling and minute volume, other discussions inherent to the outcomes from extubation might be generated. If we had analyzed the cerebral flow and its metabolism continuously,^27^ it would have been possible to have stronger support to explain the lowered level of consciousness, which was the primary cause of reintubation in these patients.

However, these resources have a high cost and are not available in all institutions. Our objective was to conduct a study that could reflect clinical practice. Through knowing the incidence and the main cause of reintubation among these patients, it will be possible to pay more attention to the assessment at the time of extubating these patients. Foresight and prevention of reintubation among postoperative patients following elective intracranial surgery will be essential for reducing the duration of mechanical ventilation, length of stay in the ICU, length of hospitalization, incidence of respiratory complications, hospital costs and mortality.

We did not find any explanation for the higher incidence of reintubation among female patients. There were no differences among reintubated patients according to gender, local topography of the surgery and disease, corroborating the literature^4^^,^^19^ (Tables 4 and 5).

**Table 4.** Distribution of reintubated patients according to gender and local topography of the surgery

Gender	Local topography of the surgery		P	Odds ratio	95% CI
	Supratentorial	Infratentorial			
Male - n (%)	4 (80)	1 (20)	0.81	3.11	[0.11; 15.71]
Female - n (%)	12 (75)	4 (25)			

CI = confidence interval.

**Table 5.** Distribution of reintubated patients according to gender and disease

Gender	Disease		P	Odds ratio	95% CI
	Tumor	Vascular			
Male - n (%)	4 (80)	1 (20)	0.6	3.11	[0.28; 34.43]
Female - n (%)	9 (56)	7 (44)			

CI = confidence interval.

## CONCLUSIONS

During the postoperative period following elective intracranial surgery, the incidence of reintubation was 12.4%. The main cause was lowering of the level of consciousness. Female gender and altered level of consciousness at the time of extubation were related to higher incidence of reintubation. Reintubation was associated with pulmonary complications, longer duration of mechanical ventilation, hospitalization and stay in the ICU, and greater incidence of tracheostomy and mortality.

## References

[1] Esteban A, Alía I, Tobin MJ (1999). Effect of spontaneous breathing trial duration on outcome of attempts to discontinue mechanical ventilation. Spanish Lung Failure Collaborative Group. Am J Respir Crit Care Med.

[2] Ely EW, Baker AM, Evans GW, Haponik EF (1999). The prognostic significance of passing a daily screen of weaning parameters. Intensive Care Med.

[3] Epstein SK, Ciubotaru RL, Wong JB (1997). Effect of failed extubation on the outcome of mechanical ventilation. Chest.

[4] Sogame LC, Vidotto MC, Jardim JR, Faresin SM (2008). Incidence and risk factors for postoperative pulmonary complications in elective intracranial surgery. J Neurosurg.

[5] Macintyre NR, Cook DJ, Ely EW (2001). Evidence-based guidelines for weaning and discontinuing ventilatory support: a collective task force facilitated by the American College of Chest Physicians; the American Association for Respiratory Care; and the American College of Critical Care Medicine. Chest.

[6] Esteban A, Anzueto A, Frutos F (2002). Characteristics and outcomes in adult patients receiving mechanical ventilation: a 28-day international study. JAMA.

[7] Demling RH, Read T, Lind LJ, Flanagan HL (1988). Incidence and morbidity of extubation failure in surgical intensive care patients. Crit Care Med.

[8] Lee PJ, MacLennan A, Naughton NN, O’Reilly M (2003). An analysis of reintubations from a quality assurance database of 152,000 cases. J Clin Anesth.

[9] Chinachoti T, Chau-in W, Suraseranivongse S, Kitsampanwong W, Kongrit P (2005). Postoperative reintubation after planned extubation in Thai Anesthesia Incidents Study (THAI Study). J Med Assoc Thai.

[10] Torres A, Gatell JM, Aznar E (1995). Re-intubation increases the risk of nosocomial pneumonia in patients needing mechanical ventilation. Am J Respir Crit Care Med.

[11] Epstein SK, Ciubotaru RL (1998). Independent effects of etiology of failure and time to reintubation on outcome for patients failing extubation. Am J Respir Crit Care Med.

[12] Vallverdú I, Calaf N, Subirana M (1998). Clinical characteristics, respiratory functional parameters, and outcome of a two-hour T-piece trial in patients weaning from mechanical ventilation. Am J Respir Crit Care Med.

[13] Pereira ED, Fernandes AL, da Silva Anção M (1999). Prospective assessment of the risk of postoperative pulmonary complications in patients submitted to upper abdominal surgery. Sao Paulo Med J.

[14] Brochard L, Rauss A, Benito S (1994). Comparison of three methods of gradual withdrawal from ventilatory support during weaning from mechanical ventilation. Am J Respir Crit Care Med.

[15] Esteban A, Frutos F, Tobin MJ (1995). A comparison of four methods of weaning patients from mechanical ventilation. Spanish Lung Failure Collaborative Group. N Engl J Med.

[16] Ely EW, Baker AM, Dunagan DP (1996). Effect on the duration of mechanical ventilation of identifying patients capable of breathing spontaneously. N Engl J Med.

[17] Esteban A, Alía I, Gordo F (1997). Extubation outcome after spontaneous breathing trials with T-tube or pressure support ventilation. The Spanish Lung Failure Collaborative Group. Am J Respir Crit Care Med.

[18] Epstein SK (2002). Decision to extubate. Intensive Care Med.

[19] Namen AM, Ely EW, Tatter SB (2001). Predictors of successful extubation in neurosurgical patients. Am J Respir Crit Care Med.

[20] Chevron V, Ménard JF, Richard JC (1998). Unplanned extubation: risk factors of development and predictive criteria for reintubation. Crit Care Med.

[21] Gil B, Frutos-Vivar F, Esteban A (2003). Deleterious effects of reintubation of mechanically ventilated patients. Clinical Pulmonary Medicine.

[22] Rothaar RC, Epstein SK (2003). Extubation failure: magnitude of the problem, impact on outcomes, and prevention. Curr Opin Crit Care.

[23] Frutos-Vivar F, Ferguson ND, Esteban A (2006). Risk factors for extubation failure in patients following a successful spontaneous breathing trial. Chest.

[24] Sogame LC, Faresin SM, Vidotto MC, Jardim JR (2008). Postoperative study of vital capacity and ventilation measurements following elective craniotomy. Sao Paulo Med J.

[25] Ingersoll GL, Grippi MA (1991). Preoperative pulmonary status and postoperative extubation outcome of patients undergoing elective cardiac surgery. Heart Lung.

[26] Daley BJ, Garcia-Perez F, Ross SE (1996). Reintubation as an outcome predictor in trauma patients. Chest.

[27] Bergsneider M, Hovda DA, Lee SM (2000). Dissociation of cerebral glucose metabolism and level of consciousness during the period of metabolic depression following human traumatic brain injury. J Neurotrauma.

